# Vitamin D receptor, vitamin D binding protein and CYP27B1 single nucleotide polymorphisms and susceptibility to viral infections in infants

**DOI:** 10.1038/s41598-021-93243-3

**Published:** 2021-07-05

**Authors:** Maria Zacharioudaki, Ippokratis Messaritakis, Emmanouil Galanakis

**Affiliations:** 1 Department of Paediatrics, School of Medicine, University of Crete, Heraklion, Greece; 2 Laboratory of Child Health, School of Medicine, University of Crete, Heraklion, Greece; 3 Laboratory of Translational Oncology, School of Medicine, University of Crete, Heraklion, Greece

**Keywords:** Genetics research, Paediatric research, Immunogenetics, Infection, Infectious diseases

## Abstract

The role of vitamin D in innate and adaptive immunity is recently under investigation. In this study we explored the potential association of genetic variances in vitamin D pathway and infections in infancy. Τhis prospective case–control study included infants 0–24 months with infection and age-matched controls. The single nucleotide polymorphisms of vitamin D receptor (*VDR*) gene (*BsmI, FokI, ApaI, TaqI*), vitamin D binding protein (VDBP) (*Gc* gene, *rs7041*, *rs4588*) and CYP27B1 (*rs10877012*) were genotyped by polymerase chain reaction-restriction fragment length polymorphism. In total 132 infants were enrolled, of whom 40 with bacterial and 52 with viral infection, and 40 healthy controls. As compared to controls, *ΤaqI* was more frequent in infants with viral infection compared to controls (p = 0.03, OR 1.96, 95% CI 1.1–3.58). Moreover, *Gc1F* was more frequent in the control group compared to infants with viral infection (p = 0.007, OR 2.7, 95% CI 1.3–5.6). No significant differences were found regarding the genetic profile for VDR and VDBP in infants with bacterial infection compared to the controls and also regarding CYP27B1 (*rs10877012*) between the studied groups. Genotypic differences suggest that vitamin D pathway might be associated with the host immune response against viral infections in infancy.

## Introduction

Infections represent a major cause of morbidity and mortality during infancy^1^. The role of vitamin D in innate and adaptive immunity and the impact on susceptibility to infections are increasingly under investigation^2–5^. The effects of vitamin D are exerted through the vitamin D receptor (VDR), which is a transcription factor, and vitamin D binding protein (VDBP) is the major plasma carrier for vitamin D^3^. Vitamin D undergoes two hydroxylation processes before the interaction with VDR on target genes; the first results in 25-hydroxyvitamin D (25[OH]D), and the second is conducted by the 1 α-hydroxylase enzyme (CYP27B1), resulting to the active metabolite 1,25-dihydroxyvitamin D (1,25[OH]2D)^3^. VDR and CYP27B1 are expressed in the majority of immune cells^3–5^. Vitamin D induces the expression of antimicrobial peptides (cathelicidin and defensine), regulates the proliferation of T cells and enhances innate immune response through interferon pathways, induction of macrophage activation, enhancement of phagocytosis and chemotaxis^3–5^. VDBP has been shown to demonstrate a direct role in innate immunity by participating in the activation of macrophages and chemotaxis^6^. It has been reported that vitamin D increases the antiviral activity of bronchial epithelial cells^6–9^. In fact, VDR and CYP27B1 are expressed in respiratory epithelial cells; RNA viruses augment the expression of CYP27B1 and thus the endogenous activation of 25-OH-vitamin D to 1,25-OH-Vitamin D in the respiratory epithelial cells with potent antiviral effects^6–9^. Moreover, Vitamin D pathway has been associated to Toll-like-receptor downregulation to which respiratory syncytial virus (RSV) is bound in respiratory epithelial cells^6–9^.

Vitamin D deficiency has been increasingly reported worldwide, even in countries with extensive sunshine^10^. Vitamin D deficiency has been associated with susceptibility to infections of the respiratory and gastrointestinal tract in school-aged children, to sepsis in children and adults and to severity and mortality of infection with the severe acute respiratory syndrome coronavirus-2 (SARS-CoV-2)^3,11–15^. There are four major SNPs of *VDR* gene (chromosome 12q13-12q14) described in the literature that are potentially functional and affect the expression of the *VDR* gene: *FokI* (*rs2228570*) G/A change in exon 2, *TaqI* (*rs731236*) T/C change in exon 9, *BsmI* (*rs1544410*) A/G and *ApaI* (*rs7975232*) G/T change in intron 8^16–18^. VDBP is encoded by single copy *Gc* gene located on chromosome 4q12-q13^19^. The two most common SNPs of *Gc* gene are *rs7041* T/G change (Asp416Glu) and *rs4588* C/A change (Thr420Lys) in exon 11 (six haplotypes are observed); the composite genotype of these two *SNPs* results in the three variants of the *Gc* gene (*rs7041*T-*rs4588*C, *rs7041*G-*rs4588*C and *rs7041*T-*rs4588*A) that encode the three major electrophoretic variants of VDBP (allozymes), termed group- specific component 1 fast (Gc1F), Gc1 slow (Gc1S) and Gc2 respectively^19–21^. Such replacement of amino acids with different electrical charge leads in slight modification of the net charge of the protein and these variants of VDBP differ in their binding affinity to vitamin D resulting in different bioavailability and circulating levels of 25[OH]D^19^. CYP27B1-1260 promoter polymorphism *rs10877012* is located on chromosome 12q13.1-13.3^22^. The purpose of this study was to investigate the role of genetic variances in vitamin D pathway, SNPs of the receptor VDR, the main plasma carrier VDBP and the enzyme CYP27B1 in the host defense against infections during infancy. Up to date data regarding the role of vitamin D pathway in susceptibility to infections in this age group are limited.

## Methods

### Study population and single nucleotide polymorphisms selection

This prospective case–control study was conducted in the Department of Paediatrics in a tertiary hospital, the University Hospital of Heraklion. The study included otherwise healthy infants aged 0–24 months that were hospitalized due to either bacterial or viral infection (cases) or other reasons (controls). Infants with bacterial infection had either confirmation by positive culture or clinically diagnosed bacterial infection (fever, site of infection, imaging and laboratory findings indicative of bacterial infection). Infants with viral infection had compatible clinical and laboratory findings. The controls were healthy infants that were hospitalized for other non-infectious reasons, for example accidents. Exclusion criteria were age > 24 months of age, prematurity defined as gestational age < 36 weeks and diagnosis of comorbidities that would predispose to infections (i.e., congenital heart disease, multiple congenital anomalies inherited or secondary immune defects causing immunosuppression, and chronic pulmonary or upper airway disease). The control group had no history of hospitalization due to infection during the first two years of life. All patients that were enrolled, cases and controls, were Caucasians. The epidemiological and clinical data of all enrolled patients were recorded. The sample size was determined using recent literature data and prospective study population calculation program (G* Power 3.1.6 and Power and Sample Size Program).

The single nucleotide polymorphisms (SNPs) that were selected to be studied were those already investigated in association studies between *VDR* gene, *VDBP*, *CYP27B1* gene and a diverse range of phenotypes. The SNPs that were studied were *FokI, BsmI, ApaI, TaqI *(*VDR*); *rs7041* and *rs4588* (*Gc*) and the *CYP27B1-1260* promoter polymorphism (*rs10877012*).

### Genotyping and SNPs investigation

Peripheral whole blood samples were collected in tubes containing EDTA during standard investigation and stored at − 20 °C. DNA was extracted from leukocytes using a DNA extraction kit (QIAamp DNA mini kit; Qiagen, Hilden, Germany) according to the manufacturer’s protocol and stored at − 20 °C. NanoDrop ND-1000, version 3.3 (ThermoFisher Scientific, Waltham, MA, USA) was used for DNA quantification. Polymorphisms were genotyped by DNA amplification with polymerase chain reaction (PCR) and sequence-specific oli-gonucleotide primers for amplification of selected SNPs followed by the RFLP (Supplementary Table). PCR cycling conditions for all the SNPs that were studied are described in Table 1. The amplified products were digested using restriction enzymes; *FokI*, *BsmI*, *ApaI* and *TaqI* (all Takara, United States) for the VDR gene, *StyI* and *HaeIII* (Thermo Fisher Scientific | Waltham, United States) for *rs4588* and *rs7041* of the *Gc* gene respectively and *Pfel* (Thermo Fisher Scientific) for *rs10877012* of *CYP27B1-1260* promoter polymorphism, according to manufacturer's instructions. The restriction fragments were separated by electrophoresis on a 2.5% agarose gel, stained with SYBR^®^ Safe DNA Gel Stain (Invitrogen, Thermo Fisher Scientific) and visualized with a 312-nm ultraviolet transilluminator. Genotypes were designed by a lowercase letter for the presence of the restriction site and by a capital letter for its absence.

**Table 1:** PCR amplification conditions for each studied SNP.

SNP	PCR amplification conditions
*VDR* *ApaI, TaqI*	Initial heating at 94 °C for 3 min 35 cycles of: 94 °C for 30 s, 59 °C for 30 s, 72 °C for 30 s Final extension step at 94 °C 10 min
*VDR* *BsmI*	Initial heating at 94 °C 3 min 35 cycles of: 94 °C for 30 s, 57 °C for 30 s, 72 °C for 30 s Final extension step at 72 °C for 10 min
*VDR* *FokI*	Initial heating at 94 °C 3 min 36 cycles of: 94 °C for 30 s, 58 °C for 30 s, 72 °C for 30 s Final extension step at 72 °C for 10 min
*Gc (VDBP)* *rs7041, rs4588*	Initial heating at 95 °C for 15 min 35 cycles of: 94 °C for 45 s, 51 °C for 45 s, 72 °C for 45 s Final extension step at 72 °C for 7 min
*CYP27B1-1260 promoter rs10877012*	Initial heating at 94 °C for 5 min 30 cycles of: 94 °C for 45 s, 58 °C for 45 s, 72 °C for 45 s Final extension step at 72 °C for 7 min

### Statistical analysis

Collected study data were recorded in Excel (Microsoft, Redmond, Washington, USA). The allele frequencies for the SNPs of the VDR gene (*FokI, BsmI, ApaI *and* TaqI*) and *rs10877012* of *CYP27B1-1260* promoter polymorphism were compared between cases (infants with bacterial or viral infection) and controls. Regarding VDBP the statistical analysis was conducted after the construction of the haplotypes and the corresponding VDBP genotypes from the combination of the *rs4588* and *rs7041* polymorphisms of the *Gc* gene. Analysis was based on contingency tables, including calculations of odds ratio (OR) and of the lower and upper limits of the 95% confidence interval. Comparison of categorical variables was conducted using two-tailed Fisher's exact test. The *p* < 0.05 was considered to be the level of significance. Bonferroni correction was also applied in the results and no false positive result was revealed.

### Ethics

The study was approved by the ethics committee and the institutional review board of the University Hospital of Heraklion (4698/17-06-2015). Written informed consents were obtained from the parents of the patients before enrollment. All methods of this study were carried out in accordance with relevant guidelines and regulations.

## Results

In total 132 infants, 40 (19 males) with bacterial and 52 (30 males) with viral infection, and 40 (22 males) healthy controls were enrolled. All patients that were enrolled, cases and controls, were Greeks. The viral infection group included 40 infants with acute viral respiratory tract infection (acute bronchiolitis 34, of whom 19 due to RSV, and 6 with febrile viral upper respiratory infection), 6 cases with viral gastroenteritis and 6 with febrile viral infection. The bacterial infection group included infants that were hospitalized due to urinary tract infection (23), bacterial pneumonia (7), meningitis-bacteremia (1), acute bacterial otitis media (6, of whom 2 with mastoiditis), upper respiratory tract infection (2) and staphylococcal scalded skin syndrome (1). Cases and controls did not differ significantly with respect to age or sex distribution.

### Vitamin D receptor (VDR)

*ApaI a* allele, *BsmI b* allele, *FokI f* allele and *TaqI t* allele frequencies were investigated in all 132 patients (Table 2). *ΤaqI* polymorphism, *t* allele, was more frequent in infants with viral infection compared to controls (*p* = 0.03, OR 1.96 95% CI 1.1–3.58). Moreover, *t* allele was more frequent in infants with acute viral respiratory tract infection compared to controls (*p* = 0.025, OR 2.16, 95% CI 1.15–4.10). However, no significant difference was found regarding *TaqI* distribution between infants with bacterial infections compared to the control group. No significant difference was observed regarding allele frequencies of *BsmI, FokI, ApaI* between infants with viral infection compared to controls neither between infants with bacterial infection compared to the control group (Table 2).

**Table 2:** Allele frequencies of *VDR* gene between infants with viral infection compared to controls and between infants with bacterial infection and controls.

SNP alleles	Controls N = 40	Viral N = 52	OR (95% CI); *p* value Viral vs control	Bacterial N = 40	OR (95% CI); *p* value Control vs bacterial
*TaqI* T	53 (66.3%)	52 (50%)	1.96 (1.1, 3.58); *p* = 0.03	52 (65%)	1.05 (0.55, 2.02); *p* = 0.99
*TaqI* t	27 (33.7%)	52 (50%)		28 (35%)	
*FokI* F	53 (66.3%)	64 (61.5%)	1.23 (0.67, 2.25); *p* = 0.53	51 (63.8%)	1.12 (0.58, 2.14); *p* = 0.86
*FokI* f	27 (33.7%)	40 (38.5%)		29 (36.2%)	
*BsmI* B	36 (45%)	43 (41.3%)	1.16 (0.64, 2.09); *p* = 0.65	34 (42.5%)	1.1 (0.59, 2.07); *p* = 0.87
*BsmI* b	44 (55%)	61 (58.7%)		46 (57.5%)	
*ApaI* A	41 (51.3%)	59 (56.7%)	0.8 (0.44, 1.43); *p* = 0.55	43 (53.8%)	0.9 (0.49, 1.69); *p* = 0.87
*ApaI* a	39 (48.7%)	45 (43.3%)		37 (46.2%)	

### Vitamin D binding protein (VDBP)—*Gc* gene

The two polymorphisms of the gene encoding VDBP, *Gc* gene, *rs7041* and *rs4588,* were determined in the 132 infants. The composite genotype of these two SNPs results in the three major electrophoretic variants of VDBP: Gc1F, Gc1S and Gc2. The allele frequencies of the *Gc* gene between the studied groups are presented in Table 3. Haplotype *Gc1F* of VDBP (*rs7041*T-*rs4588*C) was significantly more frequent in the control group compared to infants with viral infection (*p* = 0.007, OR 2.7, 95% CI 1.3–5.6). Moreover, *Gc1F* was more frequent in infants in the control group compared to infants with acute viral respiratory tract infection (subgroup of infants with viral infection) (*p* = 0.10, OR 3.04, 95% CI 1.34–6.93). Frequency of *Gc1F* was not significantly different between infants with bacterial infection and controls, as was not frequency of *Gc2* and *Gc1S* variants between the studied groups.

**Table 3:** Allele frequencies of *Gc* gene between infants with viral infection compared to controls and between infants with bacterial infection and controls.

*Gc* Allele Frequency	Controls N = 40	Viral N = 52	OR (95%CI); *p* value Control vs Viral	Bacterial N = 40	OR (95%CI); *p* value Control vs Bacterial
*Gc1F*	25	15	2.7 (1.3, 5.6); *p* = 0.007	16	2.5 (1.01, 6.14); *p* = 0.07
*Gc1S*	34	58	0.58 (0.3, 1.05); p = 0.1	38	0.82 (0.44, 1.52), *p* = 0.63
*Gc2*	21	31	0.83 (0.43, 1.6); p = 0.62	26	0.73 (0.37, 1.46)*, p* = 0.49

### CYP27B1

No significant difference was observed regarding allele frequencies of the *CYP27B1-1260* promoter polymorphism between controls and infants with viral infection or infants with bacterial infection.

## Discussion

We demonstrated that polymorphisms of the *VDR* gene, in particular *TaqI*, are associated with viral infections and in particular with viral respiratory tract infections, in infants. As all infants were hospitalized, vitamin D pathway may also be associated with the severity of viral infections. VDR SNPs have been associated with severe RSV bronchiolitis and vitamin D pathway has been related to the severity of SARS-CoV-2 infection^15,23^. *TaqI* has been previously linked to susceptibility to lower respiratory tract infections in early childhood and to tuberculosis in adults^16,24^. Furthermore, VDR SNPs have been associated with community acquired pneumonia in children and with *S. aureus* nasal carriage in patients with type I diabetes^25,26^. In contrast, in a case control study no significant difference was confirmed regarding the distribution of *TaqI* and *ApaI* in children with acute lower respiratory tract infection and controls^27^. *ApaI* and *BsmI* have been associated with urinary tract infections in children; however, no difference was observed in our study regarding their frequency between infants with bacterial infection and controls^28^.

In our study, *TaqI* polymorphism was more frequent in infants hospitalized with acute viral respiratory tract infections than in controls. Roth et al. similarly reported that *FokI* and *TaqI* polymorphisms are associated with viral bronchiolitis in early childhood^16^. *TaqI* may alter VDR gene expression, VDR protein structure and binding specificity for vitamin D, resulting in reduced vitamin D-related signaling pathways activity in target cells^17,29^. VDR is expressed in the majority of innate immune cells (macrophages, monocytes) and Vitamin D enhances the innate immune response, thus *TaqI* may reduce the immunomodulatory effects of Vitamin D^3–5,17,29^. The results of the present study are also supported by in vitro studies demonstrating that respiratory epithelial cells have VDR and vitamin D enhances their antiviral response, especially against RNA viruses^7–9^. These literature data explain the findings of our study since *TaqI* may contribute to the susceptibility to viral infections and especially viral respiratory tract infections in infants.

Ιn the present study, *Gc1F* was significantly more frequent in the control group compared to infants with viral infections and compared to infants with acute viral respiratory tract infections. Gc1F variant of VDBP has been reported to have greater affinity for vitamin D, potentially leading to more efficient delivery to target tissues^30–32^. Additionally, *Gc1F* has been associated to higher circulating levels of vitamin D and better response to supplementation compared to *Gc2* and *Gc1S*^21,31–34^. VDBP SNPs have been associated with susceptibility to RSV bronchiolitis in infants and to hepatitis C in adults^6,35^. VDBP is transformed by T and B lymphocytes to a potent macrophage activating factor, the Gc-MAF^19,36,37^. Interestingly, the highest activity of the Gc-MAF is reported with *Gc1F* haplotype of VDBP^37^. The aforementioned studies support the results of our study since *Gc1F* was more frequent in the control group compared to infants with viral infections and may explain the protective effect that may confer against viral infections since *Gc1F* may enhance the host response against viral infections.

CYP27B1 is expressed in macrophages, dendritic, T and B cells and *CYP27B1-1260* promoter polymorphism (*rs10877012*) has been reported to influence the levels of 1,25[OH]2D in serum and associated with HBV infection and autoimmune diseases in adults^5,38,39^. However, in our study no significant difference was observed regarding *CYP27B1-1260 p*romoter polymorphism among the studied groups.

Innate immunity is crucial in the response to viral infections. On the other hand, immune response to bacterial infections is based more in adaptive humoral immunity. VDR and CYP27B1 are expressed in the majority of innate immune cells and vitamin D enhances innate immune response through interferon pathways, induction of macrophage activation, enhancement of phagocytosis and chemotaxis^3–5^. Vitamin D also induces the expression of cathelicidin that has antiviral activity^3–5^. Moreover, VDBP participates directly in the activation of macrophages and chemotaxis^6,19^. Furthermore, it has been reported that VDR and CYP27B1 are expressed in respiratory epithelial cells with potent antiviral effects through the action of Vitamin D^6–9^. Therefore, the Vitamin D pathway demonstrates its immunomodulatory effects mostly by enhancing the innate immune response and promoting the host defense against viral infections. This explains the findings of our study since genetic differences regarding VDR and VDBP were associated with viral infections. Our viral infection group was consisted in the majority of viral respiratory tract infections; thus, our findings may be attributed to the role of the vitamin D pathway both in viral infections in general and, in particular, in viral respiratory tract infections^3–9^.

To the best of our knowledge, this is the first case control study that was conducted in infants and investigated simultaneously genetic variances in the three most important elements of Vitamin D pathway: vitamin D receptor, vitamin D binding protein that is the main plasma carrier and the enzyme of endogenous activation CYP27B1, and their association to infections. Up to date data regarding the role of vitamin D pathway in susceptibility to infections in this age group are limited. Hence, the findings of our study in this age group are innovative and of great importance since in infancy infections represent a major cause of morbidity and mortality^1^. Finally, our findings further elucidate genetic susceptibility to viral infections and may lead to the design of preventive measures or personalized medical approach in order to promote infants’ health and decrease morbidity and mortality due to infections in infancy, especially in the era of the SARS-CoV-2 pandemic.

Our findings suggest that future study of the role of vitamin D in susceptibility to viral infection in infants needs to include also levels of VDBP and vitamin D, in conjunction with the genetic profile of VDBP and VDR, to further understand the role of the vitamin D pathway in viral infections. The major limitation of the study is the small sample size; however, to overcome such a limitation, there was an age-matching of the enrolled control subjects with the patients involved. Our results need to be confirmed in larger patients’ cohort. Moreover, in our study infections were not all analyzed by causative pathogen and the group of bacterial infections was heterogenous.

## Conclusions

In this study we demonstrated that genetic variances in vitamin D pathway may modulate susceptibility to and severity of viral infections, in particular of viral respiratory tract infections, in infancy. *TaqI* was significantly more frequent in infants with acute viral infections compared to controls and *Gc1F* was more frequent in the control group compared to infants with acute viral infections. Our findings further elucidate genetic susceptibility to viral infections and detection of VDR and VDBP status might help determine high-risk infants.

## Supplementary Information

Supplementary Table.
