# Supplementary material for: Vitamin D receptor, vitamin D binding protein and CYP27B1 single nucleotide polymorphisms and susceptibility to viral infections in infants
Source: Sci Rep. 2021 Jul 5;11:13835. doi: 10.1038/s41598-021-93243-3 (PMC8257681; doi:10.1038/s41598-021-93243-3)
Supplement: Supplementary file 1 — Supplementary Table. [file 41598_2021_93243_MOESM1_ESM.docx]

**Vitamin D receptor, vitamin D binding protein and CYP27B1 single nucleotide polymorphisms and susceptibility to viral infections in infants**

**Maria Zacharioudaki^1,2^, Ippokratis Messaritakis^3^, Emmanouil Galanakis*^1,2^**

**1: Department of Paediatrics, Heraklion University Hospital, Greece**

**2: Laboratory of Child Health, School of Medicine, University of Crete, Greece**

**3: Laboratory of Translational Oncology, School of Medicine, University of Crete, Greece**

**Supplementary Table:** PCR primers designed to amplify fragments harboring VDR (*TaqI, BsmI, FokI, ApaI)*; VDBP (*rs4588, rs7041*) and CYP27B1 (*rs10877012*) SNPs

| **SNP** | **Primer** | **Sequence (5΄-> 3΄)** | **Fragment size** |
| --- | --- | --- | --- |
| *ApaI* | Forward  Reverse | CAGAGCATGGACAGGGAGCAA  GCAACTCCTCATGGCTGAGGTCTC | 740bp uncleaved,  530bp, 210bp |
| *BsmI* | Forward  Reverse | CAACCAAGACTACAAGTACCGCGTCAGTGA  AACCAGCGGGAAGAGGTCAAGGG | 825bp uncleaved,  650bp, 175 bp |
| *FokI* | Forward  Reverse | AGCTGGCCCTGGCACTGACTCTGCTCT  ATGGAAACACCTTGCTTCTTCTCCCTC | 265 bp uncleaved,  196bp, 69bp |
| *TaqI* | Forward  Reverse | CAGAGCATGGACAGGGAGCAA  GCAACTCCTCATGGCTGAGGTCTC | 740bp uncleaved, 495bp, 245 bp or 290 bp, 205 bp |
| *Gc rs7041*  *(HaeIII)* | Forward  Reverse | AAATAATGAGCAAATGAAAGAAGAC  CAATAACAGCAAAGAAATGAGTAGA | 483 bp uncleaved,  297 bp, 186 bp |
| *Gc rs4588*  *(StyI)* | Forward  Reverse | AAATAATGAGCAAATGAAAGAAGAC  CAATAACAGCAAAGAAATGAGTAGA | 483 bp uncleaved,  305 bp, 178 bp |
| *CYP27B1 rs10877012*  *(Pfel)* | Forward  Reverse | GTGTTCCCTAAGTGTTGTCTC  GCTGACTCGGTCTCCTCTG | 666 bp uncleaved,  490 bp, 176 bp |
